# A Multi-Attribute Decision-Making Approach for Critical Node Identification in Complex Networks

**DOI:** 10.3390/e26121075

**Published:** 2024-12-09

**Authors:** Xinyun Zhao, Yongheng Zhang, Qingying Zhai, Jinrui Zhang, Lanlan Qi

**Affiliations:** 1 Electronic Engineering Institute, National University of Defense Technology, Hefei 230037, China; zhaoxinyun66@nudt.edu.cn (X.Z.); zhangyongheng@nudt.edu.cn (Y.Z.); 2 Institute of Mathematics, Hefei University of Technology, Hefei 230601, China; 2023180278@mail.hfut.edu.cn (Q.Z.); 2023180284@mail.hfut.edu.cn (J.Z.)

**Keywords:** complex networks, critical node identification, node importance indicator, multi-attribute decision making

## Abstract

Correctly identifying influential nodes in a complex network and implementing targeted protection measures can significantly enhance the overall security of the network. Currently, indicators such as degree centrality, closeness centrality, betweenness centrality, H-index, and K-shell are commonly used to measure node influence. Although these indicators can identify critical nodes to some extent, they often consider node attributes from a narrow perspective and have certain limitations. Therefore, evaluating the importance of nodes using most existing indicators remains incomplete. In this paper, we propose the multi-attribute CRITIC-TOPSIS network decision indicator, or MCTNDI, which integrates closeness centrality, betweenness centrality, H-index, and network constraint coefficients to identify critical nodes in a network. This indicator combines information from multiple perspectives, including local neighborhood importance, network topological location, path centrality, and node mutual information, thereby solving the issue of the one-sided perspective of single indicators and providing a more comprehensive measure of node importance. Additionally, MCTNDI is validated through the analysis of several real-world networks, including the Contiguous USA network, Dolphins network, USAir97 network, and Tech-routers-rf network. The validation is conducted from four aspects: the results of simulated network attacks, the distribution of node importance, the monotonicity of rankings, and the similarity of indicators, illustrating MCTNDI’s effectiveness in real networks.

## 1. Introduction

Large-scale network services and data processing depend on complex network architectures [1]. Complex networks simplify intricate systems by abstracting them into nodes and edges. Their small-world [2], scale-free [3], and community-structured [4] properties have made them a focal point in fields such as sociology, biology, and computer science [5,6,7,8,9]. Nodes that are more susceptible to infection and can further infect a large number of other nodes in the network are considered critical nodes [10]. Critical nodes are essential for identifying key cities to promote sustainable development [11], protecting important transportation hubs [12,13], controlling the spread of rumors and social opinions [14], and resisting cyber attacks [15].

Critical node discovery techniques can be categorized into two types of identification methods: those based on network destruction resistance and those based on node influence [16]. The network destruction resistance method posits that the importance of a node is positively correlated with the extent of network damage caused by its removal. Since this approach alters the network’s topology during analysis, we use the second method to study the critical node identification problem. The node influence method considers the prominence of a node as a direct reflection of its importance and typically uses node importance indicators to measure the significance of a node. Common node importance indicators include degree centrality [17], closeness centrality [18], betweenness centrality [19], H-index [20], K-shell [21], etc. However, these indicators have certain limitations.

While the above indicators can evaluate node importance from specific perspectives, relying on a single indicator often provides an incomplete assessment due to the network’s diverse structural characteristics. Degree centrality and H-index are based on the attributes of a node’s neighbors, and they only consider the local structure around the node. Betweenness centrality evaluates node importance based on the shortest path information in the network. While this method takes the global structure into account, information transfer in the network may not always follow the shortest paths. Closeness centrality is based on the distance of a node from other nodes in the network. However, this method suffers from a lack of local information and cannot adequately reflect the influence of a node in the local environment, especially when the network has an obvious community structure. Although the K-shell considers the global hierarchy of the network, most nodes are distributed in the same layer after applying the method, making it challenging to distinguish the importance of nodes within the same layer.

**Figure 1:**
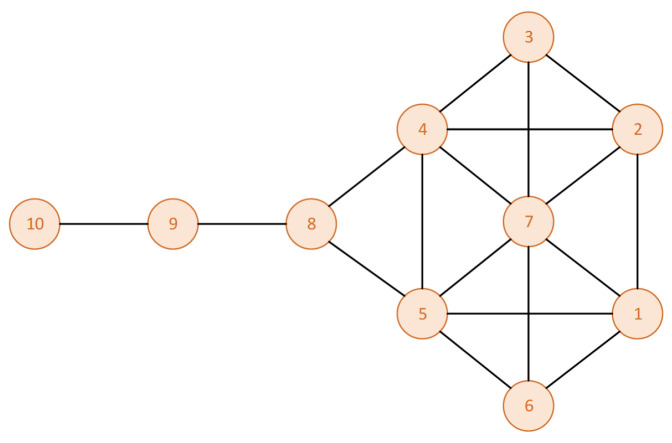
An example of a kite network.

In addition, different indicators may produce varying ranking results, making node ranking decisions challenging. Krackhardt [22] designed a kite network with ten nodes, as shown in Figure 1. In the kite network, nodes 4 and 5 have the highest closeness centrality values, and node 7 has the highest degree. Despite having a degree of only 3, node 8 possesses the lowest network constraint coefficient and betweenness centrality values in the network, indicating its crucial position in the information flow. The values of each common indicator for the kite network are shown in Table 1 (degree centrality, betweenness centrality, closeness centrality, K-shell, H-index, network constraint coefficient). From the table, it can be seen that using a single indicator may lead to homogeneous results or conflicting results under different indicators, making it difficult to differentiate between nodes for decision making.

To measure the criticality of nodes more comprehensively, a fusion of diverse node attributes needs to be considered. The multi-attribute decision-making method aims to utilize multiple indicators to more thoroughly assess the importance of nodes in the network, thereby solving the issue of incomplete assessment results associated with single indicators [23]. Integrating information from various aspects of a node’s attributes provides a comprehensive understanding of its role in the network structure, enhancing recognition accuracy and yielding a more precise ranking of node importance.

In this paper, after investigating a large amount of related work and analyzing the limitations of existing commonly used methods, we propose the multi-attribute CRITIC-TOPSIS network decision indicator, or MCTNDI. This indicator enables the integration of multiple node attribute information without incorporating subjective factors and evaluates the importance of the solution to be selected based on the inherent characteristics of the scoring data. We use existing representative node importance indicators as sub-indicators, obtain node scores using MCTNDI, and derive their importance rankings. The main contributions are as follows:

- We use closeness centrality, betweenness centrality, H-index, and network constraint coefficient as sub-indicators for multi-attribute decision making from four independent perspectives: neighborhood centrality, network topological location, path centrality, and node mutual information, taking into account multi-perspective information.
- We propose a multi-attribute decision-making indicator that combines the CRITIC and TOPSIS methods to effectively identify the sequence of critical nodes in a complex network based on its topology.
- We analyze the effectiveness of MCTNDI using simulated network attacks and complement it with evaluations of monotonicity, stability, and correlation. By comparing it with other commonly used node importance indicators, the effectiveness of MCTNDI in identifying critical nodes is verified in multiple dimensions.

This paper is structured in the following sections. In Section 2, we review related work on critical node identification. Section 3 focuses on common node importance indicators as well as the framework and process of MCTNDI. In Section 4, we design four experiments to answer four research questions related to MCTNDI. Section 5 summarizes the entire work and explores future research directions.

## 2. Related Work

In this section, we review two types of current methods for ranking the importance of critical nodes: (1) methods based on network destruction resistance and (2) methods based on node influence.

### 2.1. Identification Method Based on Network Destruction Resistance

This method utilizes the concept of “damage mapping importance” from system engineering, which reflects the importance of a node by measuring the degree of damage to network connectivity caused by its removal. Wang et al. [24] used different attack patterns to attack nodes in the power communication data and transmission network. They found that attacking important nodes had a greater impact on the invulnerability performance of the power communication network compared to random attacks, as measured by the change in network efficiency. Wang et al. [25] characterized the combat network using task chains and proposed a C2 network destruction resistance method based on task link efficiency and entropy. This method effectively represents the efficiency of information transmission and the robustness of the network topology. Qi et al. [26] proposed a quantitative resilience assessment model for metro networks. By interrupting general stations, interrupting interchange stations, and blocking traffic tunnels, they analyzed the robustness and vulnerability of metro networks under node interruptions and edge failures. Some of these methods suffer from the defect that when the removed nodes reach a certain size, the whole network is paralyzed and it is impossible to compare the importance of the relevant nodes.

### 2.2. Identification Method Based on Node Influence

This method evaluates the importance of nodes in the network from several aspects of network topological properties. However, most research methods assess nodes based on only a few aspects, making achieving comprehensive and accurate evaluation results difficult. The related approach is based on the premise of ensuring the integrity of the network without disrupting its connectivity. Depending on the focus on network properties, this approach can be further categorized into four subcategories.

Critical node identification method based on node neighborhood centrality. The idea of the method is that the influence of a node in the network is closely related to its neighboring nodes. Freeman [27] first proposed the concept of degree centrality, which posits that the more neighbors a node has, the greater its contribution to the network. Xu et al. [28] developed a critical node identification method based on the entropy of node neighboring information, which has a high correlation with degree centrality and can be applied to directed weighted networks. Chen et al. [29] designed a semi-local centrality indicator, which strikes a balance between degree centrality and other time-consuming methods. Lü et al. [20] discovered a correlation between the H-index, degree centrality, and core centrality. This type of approach only considers the structure of the local area around the node.

Critical node identification method based on network topological location. The idea of the method is that the importance of a node is closely related to its position in the network topology. The K-shell of Kitsak et al. [21] uses a recursive strategy to strip the network layer by layer of nodes whose degree does not exceed K, forming a hierarchical structure from the periphery to the core of the network. In addition to degree centrality, Freeman [27] proposed closeness centrality by calculating the distance from a node to every other node to measure its proximity to the center of the network. Wei et al. [30] considered an edge weighting method that increases the degree of the nodes at both ends of the connected edges. For the constructed weighted network, they designed a weighted K-shell decomposition method (WKs). This type of approach lacks consideration of local information and performs poorly when the network has a distinct community structure.

Critical node identification method based on path centrality. The idea of this method is that the importance of a node is closely related to the length of the path in which the node is located. Freeman proposed the method of betweenness centrality in [31], which asserts that the more shortest paths pass through a node, the more important the node is. Liu et al. [32] used harmonic centrality to find that air–rail intermodal transportation between hub airports and high-speed rail stations has a strong complementary effect on international flights. Li et al. [33] considered the resistance distance, which can more fully reflect the cost of communication between nodes, and designed a set of resistance centrality measures. Then, they proposed a method called RCWTA to identify influential nodes, where resistive harmonic centrality performed the best. This type of approach considers shortest path information, but information transfer may not always be along the shortest path.

Critical node identification method based on node mutual information. The idea of this method is to assess the importance of nodes by comprehensively analyzing the number and quality of neighboring nodes and using an iterative solution. Brin et al. [34] proposed the PageRank algorithm based on the stochastic wandering model, which posits that the importance of a web page depends on the number and quality of links from other important pages. Bonacich [35] introduced eigenvector centrality by iterating the influence of the neighbors of a node in a network. This approach ignores the role of local communities. Poulin et al. [36] used a cumulative nomination scheme, which assumes that core nodes will be nominated more frequently by other nodes and evaluates the importance of a node based on the number of nominations.

## 3. Multi-Attribute Decision-Making Approach—MCTNDI

In this section, we will introduce the framework of MCTNDI, its sub-indicators, and its process.

### 3.1. Framework of MCTNDI

Within the framework of multi-attribute decision making, the CRITIC method [37] was selected to ensure an objective and scientifically rigorous distribution of weights. In complex networks, indicators often exhibit varying levels of importance and potential interdependencies, such as correlations or disparities. Therefore, a data-driven approach is essential to accurately assess the significance of each indicator. The CRITIC method evaluates each indicator’s contribution by integrating two key aspects: the dispersion of attribute values (contrast intensity) and the correlations among indicators (conflict intensity). Contrast intensity, measured by the standard deviation of an indicator’s values, assigns higher weights to indicators with greater variability. Conflict intensity, which quantifies the independence of indicators using correlation coefficients, reduces weights for highly correlated indicators. By synthesizing these factors, the CRITIC method minimizes human bias and ensures a scientifically rational weight assignment.

To integrate the evaluation scores of various sub-indicators, the TOPSIS method [38] was employed. Identifying critical nodes in complex networks requires a multi-indicator evaluation approach, and TOPSIS consolidates multiple attributes into a single comprehensive score by comparing each node’s proximity to ideal solutions. The core principle of TOPSIS is to rank nodes based on their closeness to the positive ideal solution (representing the best indicator values) and their distance from the negative ideal solution (representing the worst indicator values). By calculating each node’s relative distances to these ideal solutions, the TOPSIS method incorporates the effects of different indicator weights and provides a prioritized ranking of node importance across multiple attributes.

**Figure 2:**
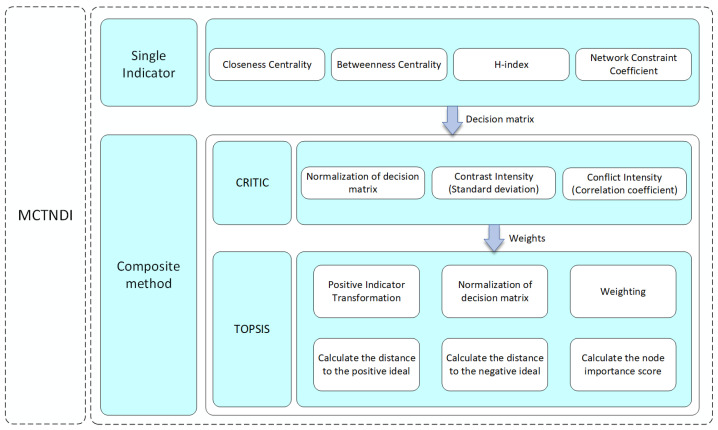
The framework of MCTNDI.

The framework of MCTNDI is shown in Figure 2. The idea of MCTNDI is to integrate node importance information from multiple dimensions and then accurately identify critical nodes in the network. In this study, we selected sub-indicators for the multi-attribute decision-making approach from four independent perspectives: the H-index, which reflects neighborhood centrality; closeness centrality, which expresses the topological location of the network; betweenness centrality, which describes path centrality; and the network constraint coefficient, which is based on the amount of mutual information between nodes. Next, the decision matrix is constructed based on the results obtained from the above indicators, and the weights of these indicators are calculated using the CRITIC method. Then, within the TOPSIS method, the positive-transformed and normalized decision matrix is weighted using the weights obtained above. TOPSIS evaluates the distance of each node from the positive ideal solution and the negative ideal solution. By calculating the relative closeness, a score is assigned to each network node. The importance ranking of all nodes is then generated based on these scores.

### 3.2. Selection and Formal Definition of Sub-Indicators

In the process of selecting sub-indicators for multi-attribute decision making, it is crucial to minimize homogeneity among the indicators. The sub-indicators should describe the attributes of nodes from different perspectives to fully reflect the information about the importance of nodes. The local information of a node is sufficient to reflect its influence within its immediate neighborhood and helps to understand the node’s function in a particular community or sub-network. If a node is at the core of a network, it is likely to be more influential. The extent of a node’s centrality in the network can be measured by analyzing the node’s topological position and calculating its distance from the network’s center. In complex networks, information generally propagates along the shortest paths. Nodes that act as mediators on these shortest paths more frequently tend to have more information. Additionally, nodes that connect different communities play a crucial role in the process of network information transfer and hold an important position in the network. Therefore, we select sub-indicators for the multi-attribute decision-making approach based on neighborhood centrality, network topological location, path centrality, and node mutual information. Each single indicator is formally defined below.

**Definition** **1.** *H-index (H).*

The H-index was initially used to analyze academic exchanges among scholars and has been spread to the research of various complex networks progressively. The H-index of node *i* is denoted as $H_{i}$ and defined as follows:

(1) $$H_{i}=H\left(k_{j1},k_{j2},\ldots ,k_{jk_{i}}\right)$$

where *i* is the node to be evaluated and $k_{i}$ denotes the degree of node *i*, and the degrees of its neighboring nodes are $k_{j1},k_{j2},\ldots ,k_{jk_{i}}$, respectively. For a finite set of real numbers $\left(x_{1},x_{2},\ldots ,x_{n}\right)$, define the operator *H*, which returns the integer $z=H(x_{1},x_{2},\ldots ,x_{n})>0$, where *z* represents the maximum possible integer such that there are at least *z* elements in the set $\left(x_{1},x_{2},\ldots ,x_{n}\right)$ whose values are not less than *z*. The idea of the H-index is to assess the influence of a node by considering the entire quality of its neighboring nodes.

**Definition** **2.** *Closeness Centrality (CC).*

Closeness centrality is a method that measures how close a node is to the center of the network. The closeness centrality of node *i* is denoted as $CC_{i}$ and is defined as follows:

(2) $$CC_{i}=\frac{N-1}{\sum_{j=1}^{N}d_{ij}}$$

where *i* is the node to be evaluated, *N* is the total number of nodes, and $d_{ij}$ is the shortest path length between node *i* and another node *j*. The idea of closeness centrality is that the closer a node is to the center of the network, the more influence it will have.

**Definition** **3.** *Betweenness Centrality (BC).*

Betweenness centrality is a method based on the shortest paths in the network. The betweenness centrality of node *i* is denoted as $BC_{i}$ and is defined as follows:

(3) $$BC_{i}=\left(\sum_{j\neq i\neq k}\frac{g_{jk}\left(i\right)}{g_{jk}}\right)\frac{N(N-1)}{2}$$

where *i* is the node to be evaluated, $g_{jk}$ is the number of shortest paths between any two nodes *j* and *k* in the entire network, $g_{jk}\left(i\right)$ denotes the number of shortest paths passing through node *i* between nodes *j* and *k*, and the factor $\frac{N(N-1)}{2}$ is used to normalize the betweenness centrality, where *N* is the total number of nodes. The idea of betweenness centrality is that the more frequently a node serves as a mediator on the shortest paths in the network, the more influential it is.

**Definition** **4.** *Network Constraint Coefficient (NCC).*

The network constraint coefficient proceeds from the structural hole theory [39], which suggests that there are unconnected gaps between nodes representing organizations in a social network. These gaps provide opportunities for information flow and resource access. If a node connects to these gaps, it can gain access to information between different organizations, thereby achieving a high level of importance in the network.

The network constraint coefficient of node *i* is denoted as $NCC_{i}$ and is defined as follows:

(4) $$NCC_{i}=\sum_{j}NCC_{ij}=\sum_{j}{\left(p_{ij}+\sum_{q}p_{iq}p_{qj}\right)}^{2},i\neq q\neq j$$

(5) $$p_{ij}=\frac{z_{ij}}{\sum_{q}z_{iq}}$$

where *i* is the node to be evaluated, *j* is a neighboring node of *i*, $p_{iq}$ is the proportion of resources allocated by *i* to *q*, $z_{iq}$ is the connection between *i* and *q*, $p_{ij}$ is direct connection, and $\sum_{q}p_{iq}p_{qj}$ is indirect connection. The value of $NCC_{i}$ is in the range of [0,1]. The value of the network constraint coefficient is negatively correlated with the extent of structural holes. The lower the value, the more structural holes the node occupies and the more important it is in the network.

### 3.3. Process of MCTNDI

For any undirected, unweighted complex network with *q* nodes, its node set can be denoted as $A=\{a_{1},a_{2},a_{3},\ldots ,a_{q}\}$. The node scores for this network under the H-index, closeness centrality, betweenness centrality, and network constraint coefficient indicators are calculated as described in the previous section. These scores can be represented as a set of indicators $S=\{S_{1},S_{2},S_{3},S_{4}\}$, where the value of the *n*-th importance indicator of the *m*-th node is $S_{n}\left(a_{m}\right)\left(m=1,\cdots ,q;n=1,\cdots ,4\right)$. Based on this, the following decision matrix can be constructed:

(6) $$X=\left[x_{mn}\right]=\left[\begin{array}{cccc} S_{1}\left(a_{1}\right) & S_{2}\left(a_{1}\right) & S_{3}\left(a_{1}\right) & S_{4}\left(a_{1}\right)\\ S_{1}\left(a_{2}\right) & S_{2}\left(a_{2}\right) & S_{3}\left(a_{2}\right) & S_{4}\left(a_{2}\right)\\ \vdots & \vdots & \vdots & \vdots \\ S_{1}\left(a_{q}\right) & S_{2}\left(a_{q}\right) & S_{3}\left(a_{q}\right) & S_{4}\left(a_{q}\right) \end{array}\right]$$

Since different indicators have varying value ranges, the matrix needs to be normalized. As discussed in the previous section, all indicators are benefit-type indicators except for the network constraint coefficient, which is a cost-type indicator. Therefore, the H-index, closeness centrality, and betweenness centrality are normalized using the following formula:

(7) $$r_{mn}=\frac{x_{mn}-x_{n}^{\min}}{x_{n}^{\max}-x_{n}^{\min}}$$

while network constraint coefficients are normalized using the following formula:

(8) $$r_{mn}=\frac{x_{n}^{\max}-x_{mn}}{x_{n}^{\max}-x_{n}^{\min}}$$

Next, the contrast intensity is measured by the standard deviation $\sigma_{n}$, and ${\bar{r}}_{n}$ is the average of the scores in the assessment indicator $S_{n}$:

(9) $$\sigma_{n}=\sqrt{\frac{\sum_{m=1}^{q}\left(r_{mn}-\bar{r_{n}}\right)}{q}}$$

The conflict intensity $Conflict_{n}$ between any two indicators $S_{n}$ and $S_{u}$ is measured by the Pearson correlation coefficient $\rho_{nu}$, as shown in the equation below:

(10) $$\rho_{mu}=\frac{\sum_{m=1}^{q}\left(r_{mn}-{\bar{r}}_{n}\right)\left(r_{mu}-{\bar{r}}_{u}\right)}{\sqrt{\sum_{m=1}^{q}{\left(r_{mn}-{\bar{r}}_{n}\right)}^{2}\sum_{m=1}^{q}{\left(r_{mu}-{\bar{r}}_{u}\right)}^{2}}}$$

(11) $$Conflict_{n}=\sum_{u=1}^{4}\left(1-\rho_{nu}\right)$$

The weights of each indicator are obtained through two steps: the calculation of the composite information quantity and the standardization of weights. The composite information quantity is calculated by fusing the contrast intensity and the conflict intensity. The following formula multiplies the two to obtain the composite information quantity $Q_{n}$ of the assessment indicator $S_{n}$. This product reflects the ability of each indicator to provide unique information while maintaining independence from other indicators.

(12) $$Q_{n}=\sigma_{n}\times Conflict_{n}$$

Standardized weights are obtained by scaling the *Q*-values of all indicators according to the following formula to obtain the CRITIC decision weights $\omega_{n}$, ensuring that the sum of the weights is one.

(13) $$\omega_{n}=\frac{Q_{n}}{\sum_{k=1}^{4}Q_{k}}$$

After obtaining the CRITIC weights, since the network constraint coefficients are cost-type indicators, the corresponding columns in the decision matrix are normalized using the following formula. The remaining indicators are benefit-type indicators and are not processed further.

(14) $$x_{mn}=x_{mn}^{\prime }=\max\left(x_{mn}\right)-x_{mn}$$

Then, the positively transformed decision matrix is normalized, and the matrix is weighted using the obtained weights to construct the weighted normalized decision matrix *Z*. See the following equation:

(15) $$Y=y_{mn}=\frac{x_{mn}}{\sqrt{\sum_{m=1}^{q}x_{mn}^{2}}}$$

(16) $$Z=\omega_{n}y_{mn}$$

Subsequently, the positive and negative ideal solutions are derived from it. $Vector^{+}$ is the positive ideal solution vector, and $Vector^{-}$ is the negative ideal solution vector.

(17) $$Vector^{+}=\left\{\max_{m}z_{mn}\right\}=\left\{z_{1}^{+},z_{2}^{+},\ldots ,z_{m}^{+}\right\}$$

(18) $$Vector^{-}=\left\{\min_{m}z_{mn}\right\}=\left\{z_{1}^{-},z_{2}^{-},\ldots ,z_{m}^{-}\right\}$$

The Euclidean distances from each selected target to the nearest positive ideal solution and the farthest negative ideal solution are calculated according to the following equations, respectively.

(19) $$des_{m}^{+}=\sqrt{\sum_{n=1}^{4}{\left(z_{mn}-\upsilon ector_{m}^{+}\right)}^{2}}$$

(20) $$des_{m}^{-}=\sqrt{\sum_{n=1}^{4}{\left(z_{mn}-\upsilon ector_{m}^{-}\right)}^{2}}$$

Finally, the relative closeness $c_{m}$ of each node is calculated to quantitatively evaluate the performance of the *m*-th node. The value of the relative closeness $c_{m}$ represents the importance score of the *m*-th node in the network, from which the importance ranking sequence of all nodes is generated. The higher the score of a node, the more influential it is in the network.

(21) $$c_{m}=\frac{des_{m}^{-}}{des_{m}^{-}+des_{m}^{+}}$$

## 4. Experiment

In this section, we focus on evaluating the effectiveness of MCTNDI in determining the importance of nodes and analyzing its effectiveness to identify critical nodes in complex networks. Specifically, the evaluations aim to answer the following research questions (RQs).

- RQ1: How effectively does MCTNDI perform in identifying critical nodes in complex networks?
- RQ2: Are the node importance scores obtained using MCTNDI stable? What is their distribution?
- RQ3: Does using MCTNDI result in a situation where the nodes are deemed equally important, leading to an inability to distinguish their relative importance?
- RQ4: What is the relationship between MCTNDI and commonly used indicators of node importance?

### 4.1. Experimental Setup

To evaluate MCTNDI, we use four representative experimental network datasets for analysis: the Contiguous USA network, the Dolphins network, the USAir97 network, and the Tech-routers-rf network. Each network has distinct structural characteristics, providing a diverse set of scenarios for testing MCTNDI and ensuring that the evaluation is not limited by the structure of a particular class of networks.

**Figure 3:**
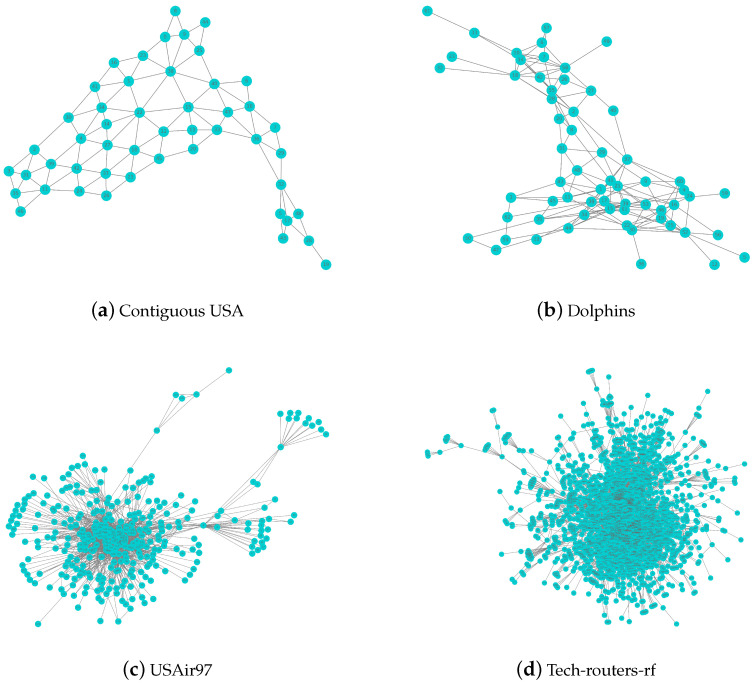
Network topology of four experimental networks.

The Contiguous USA network is a geographic boundary network that covers the 48 contiguous states in the continental United States as well as the District of Columbia. Due to their unique geographic locations, Alaska and Hawaii are not contiguous with other states and are therefore not included in this dataset. The Contiguous USA network consists of 49 nodes and 107 edges, with each node representing a state or a state-level administrative region (SAR) and each edge representing a common border between two states (or SARs). The network topology is shown in Figure 3a, which shows the sparser structure of this network.

The Dolphins network is a typical social network representing the social interactions of 62 broad-snouted dolphins in Agate Bay, New Zealand. It is often used in the analysis of complex social networks. The Dolphins network consists of 62 nodes and 159 edges, with each node representing a dolphin and each edge representing a frequent communication relationship between two dolphins. The network topology is shown in Figure 3b, and the network is roughly divided into two communities.

The USAir97 network is an infrastructure network that contains a list of all flights operated by American Airlines in 1997, representing the connections between all the airports served by the airline. The relationships between the nodes in this network are highly complex. The USAir97 network consists of 332 nodes and 2126 edges, where the nodes represent individual airports, and each edge represents a direct flight route between the airports. The topology of the network is shown in Figure 3c. The USAir97 network is more complex than the previous two networks and is suitable for analyzing the performance of MCTNDI on large and complex networks.

The Tech-routers-rf network is a technical routing network consisting of 2113 nodes and 6632 edges, where the nodes represent different routers and the edges denote connections between routers. This network shows the actual distributional architecture of a routing-level network. The network topology of Tech-routers-rf is shown in Figure 3d, which is significantly larger than the three aforementioned networks and is suitable for analyzing the performance of MCTNDI on large-scale networks.

In the experiments, we use the above datasets to compare and analyze MCTNDI with classical indicators. The comparison indicators selected for the experiments include closeness centrality, betweenness centrality, H-index, and network constraint coefficient. To enhance the referential value of the experiments, some tests also introduce degree centrality, eigenvector centrality, K-shell, and random indicator as benchmark tests for auxiliary comparisons. Comparison tests utilizing the sub-indicators of MCTNDI can demonstrate whether MCTNDI can effectively synthesize the information from these sub-indicators, provide more comprehensive assessment results, and validate the effectiveness of MCTNDI.

### 4.2. Evaluation Results

#### 4.2.1. Effectiveness Analysis of MCTNDI in Identifying Network Critical Nodes (RQ1)

In complex networks, the failure of critical nodes will bring great negative impact on the operational efficiency of the network. Simulating network attacks by removing nodes is one of the main ways to verify the effectiveness of critical node identification techniques. The idea is to assess the effectiveness of the node importance indicator based on the degree of network damage after node removal. The degree of network damage is measured by the change in network efficiency in the experiment.

In the following equations, *G* denotes the node set of the experimental network, *N* denotes the number of nodes in the experimental network, $d_{ij}$ represents the length of the shortest path between node *i* and node *j*, and NE denotes the current network efficiency.

(22) $$NE=\frac{1}{N(N-1)}\sum_{i\neq j\in G}\frac{1}{d_{ij}}$$

In the following equation, ne denotes the network efficiency of *G* after the deletion of nodes, NE denotes the initial network efficiency of *G* before the deletion of nodes, and NEP denotes the network efficiency ratio. A smaller value of NEP indicates that the damage to the network is more severe after an attack, indicating that the deleted nodes are more important.

(23) $$NEP=\frac{ne}{NE}$$

**Figure 4:**
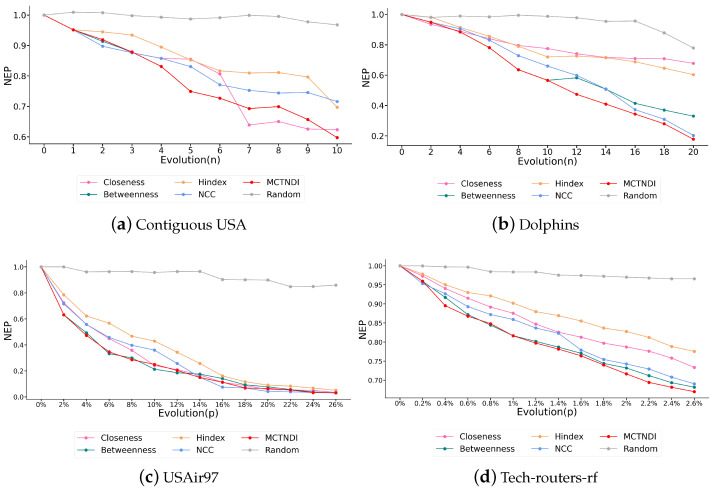
Changes in network efficiency caused by deliberate attacks in four networks using MCTNDI and different single indicators.

In experiments, we used a node-by-node or pair-by-pair removal strategy for the smaller Contiguous USA and Dolphins networks and a bulk node removal strategy for the larger USAir97 and Tech-routers-rf networks. We labeled the nodes whose MCTNDI scores ranked in the top 20% of the four experimental networks, as shown in Appendix A. During the experiment, we also introduced a random indicator for comparison. The results of attacking the networks based on the node rankings obtained from different indicators are shown in Figure 4, where the vertical axis represents the change in network efficiency before and after each round of attack. For the Contiguous USA network and the Dolphins network, the horizontal axis represents the total number of removed nodes. For the USAir97 and Tech-routers-rf networks, we scaled deliberate attacks to minimize the visual impact of small network changes due to their larger size, with the horizontal axis representing the ratio of removed nodes to the total number of nodes. According to the size of the USAir97 network and the Tech-routers-rf network, the rate of node removal in a single round was set to 2% and 0.2%, respectively.

**Figure 5:**
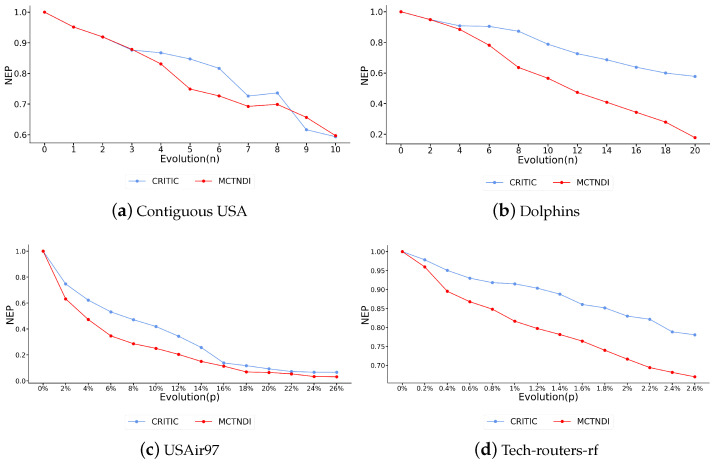
Changes in network efficiency caused by deliberate attacks in four networks using different multi-attribute methods.

We compared the MCTNDI and CRITIC methods, as shown in Figure 5, using the same sub-indicators for both. The results indicate that the network efficiency decline rate associated with MCTNDI is higher than that of the CRITIC method across the four experimental networks, demonstrating that MCTNDI is more effective in identifying critical nodes in complex networks.

The results in Figure 4 demonstrate that the low effectiveness of random attacks on real-world complex networks highlights their optimized topological configurations, which enhance overall resistance, strengthen defenses, and enable robustness under random simulated attacks. During the deliberate attacks on the Contiguous USA network and the Dolphins network, the change in network efficiency caused by MCTNDI for the top-ranked nodes is greater than that of the other indicators. The network efficiency corresponding to closeness centrality plummets when the seventh node is removed from the Contiguous USA network due to the removal of this node causing the network to be divided into two unconnected components. The network efficiency can be greatly affected by the interruption of communication between the two sub-networks. Although there are instances where the rate of decrease in network efficiency after node deletion is lower than that of individual indicators, overall, MCTNDI is effective in identifying critical nodes.

During the attack on the USAir97 network, the decline rate of network efficiency corresponding to MCTNDI is the highest in the initial phase. Although the decline rates of each indicator fluctuate slightly in the subsequent phases, MCTNDI maintains its advantage during the rapid decline phase. Meanwhile, compared to common single indicators, MCTNDI exhibits a faster network efficiency convergence rate when deliberately attacking the Tech-routers-rf network. In Figure 4c,d, when approximately 2.5% of the nodes in the network are removed, the network efficiency corresponding to MCTNDI drops below 0.7. This demonstrates the vulnerability of real networks to deliberate attacks and also reflects that MCTNDI performs well in identifying critical nodes in larger-scale networks. The figure shows that the MCTNDI curve closely overlaps with the betweenness curve in most cases. This phenomenon can be attributed to the validation of the metric’s effectiveness through network efficiency, which depends on the shortest paths between nodes. Betweenness centrality quantifies the frequency with which a node serves as an intermediary in these shortest paths, creating an inherent correlation between network efficiency and betweenness centrality. Additionally, the majority of nodes exhibit a betweenness centrality value of zero. In the computation of distances to the ideal solution in the TOPSIS method, this results in most nodes have distances that approach extreme values, potentially amplifying the ranking effects of betweenness centrality within the TOPSIS framework.

The experimental results also illustrate the limitations of some single indicators in identifying critical nodes in the network. For the H-index, it only considers the information within the node’s local neighborhood and lacks a broader perspective of the entire network, making it less effective when dealing with larger networks. For the closeness centrality, it lacks consideration of the local structure of nodes and, as mentioned before, performs poorly in Dolphins networks with an obvious community structure. In addition, the effectiveness of a single indicator in identifying critical nodes varies widely across different networks. In contrast, the network efficiency of MCTNDI decreases rapidly in all attack experiments across the four different networks, suggesting that MCTNDI is effective in identifying critical nodes in complex networks.

#### 4.2.2. Distribution Analysis of Node Importance Under Each Indicator (RQ2)

**Figure 6:**
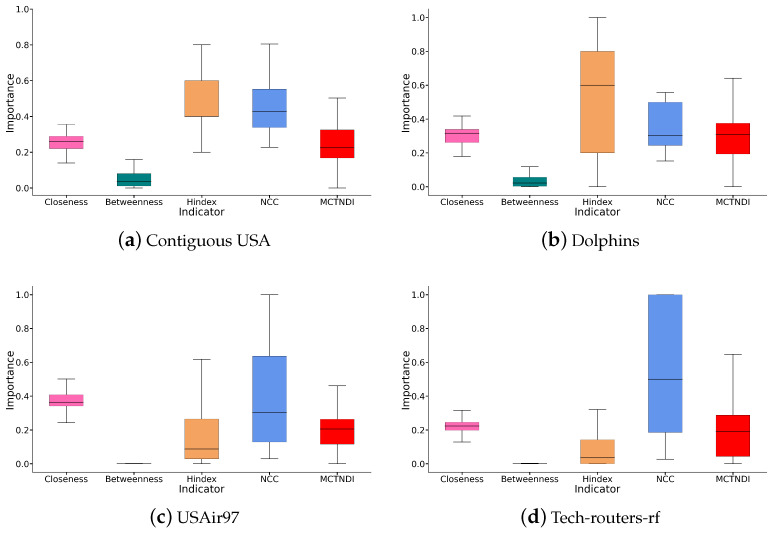
Distribution of node importance scores for four networks under different indicators.

After normalizing the scores for each indicator, we present the distribution of node importance under different indicators through a box plot in Figure 6. The horizontal axis indicates the different evaluation indicators, and the vertical axis indicates the distribution of node importance scores.

Figure 6 shows that, in terms of the distribution of node importance, the distribution range of MCTNDI’s importance scores is more concentrated and evenly distributed. This phenomenon reflects that MCTNDI has a more stable performance, reduces errors due to the randomness or irregularity of the network topology, and indicates that it considers and comprehensively analyzes multiple attributes in the calculation process.

#### 4.2.3. Monotonicity Analysis of Node Rankings Under Different Indicators (RQ3)

Ranking monotonicity of nodes is one of the key indicators for evaluating the performance of critical node identification methods. An increase in rank monotonicity means that the set of nodes with the same score in the output sequence will be smaller and the differentiation of importance among nodes will be higher. Based on Bae [40], we define the monotonicity indicator as follows:

(24) $$Monotonicity_{p}\left(I\right)={\left[1-\frac{\sum_{i\epsilon I}N_{i}\left(N_{i}-1\right)}{N_{p}\left(N_{p}-1\right)}\right]}^{2}$$

where *N* is the size of the ranking vector *I*, $N_{i}$ denotes the number of nodes with the same indicator evaluation score, $N_{p}$ denotes the number of candidate nodes selected from the target network by the ratio *p*, and the monotonicity $Monotonicity_{p}\left(I\right)$ fluctuates in the range [0,1]. This value represents the ratio of nodes with the same ranking score to the set of candidate nodes. The monotonicity $Monotonicity_{p}\left(I\right)$ is 1 if the ranking vector *I* is completely monotonic and 0 if all nodes in *I* have the same score.

**Figure 7:**
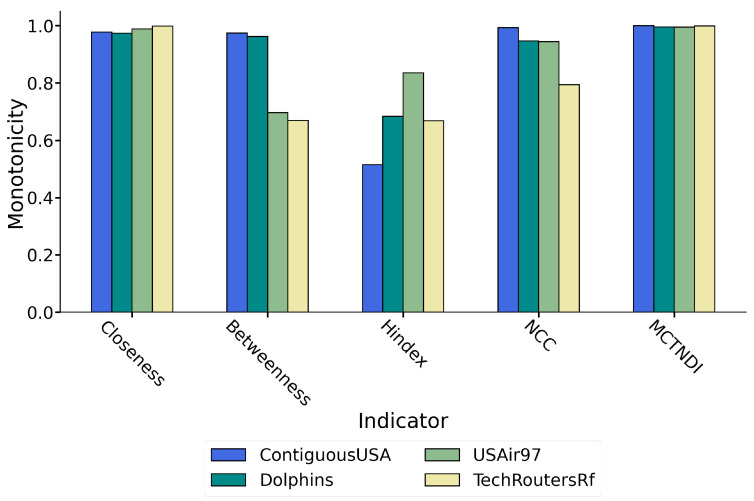
Monotonicity results of different indicators in four experimental networks.

In the experiment, we set p=1, i.e., we use all the nodes in the four experimental networks to compute the monotonicity of the entire network separately, and the results are shown in Figure 7.

Figure 7 shows that in the four experimental networks, the monotonicity of node importance rankings using MCTNDI approaches the ideal value of 1, while other common single indicators, except for closeness centrality, have significantly lower monotonicity. The composite indicator MCTNDI exhibits better monotonicity than single indicators, which helps to improve the accuracy of node importance ranking and reduces the number of nodes with the same score.

We observe that the monotonicity of the H-index is significantly lower than the other indicators. The calculation results show that in the Contiguous USA network and the Dolphins network, the H-index divides 49 nodes and 62 nodes, respectively, into only 6 gradients. There are a large number of nodes with the same scores within each gradient, which makes it unable to differentiate the importance of nodes within the same gradient.

The monotonicity of betweenness centrality in larger-scale experimental networks, such as USAir97 and Tech-routers-rf, is lower than that of other indices. This is because a significant proportion of nodes have a betweenness centrality value of zero, making it impossible to distinguish the importance of these nodes based solely on betweenness centrality. In contrast, MCTNDI effectively avoids the problem of a large number of nodes having the same importance score by integrating network topology information from multiple perspectives. The experiments demonstrate the advantages of MCTNDI in refining the ranking of critical nodes.

#### 4.2.4. Similarity Analysis of MCTNDI with Different Indicators (RQ4)

To explore the similarities and differences between MCTNDI and other single indicators in the ranking of node importance, we used Kendall’s correlation coefficient [41] to calculate the similarity between the indicators. Kendall’s coefficient τ is a reliable tool for correlation analysis and is defined as shown in the following equation:

(25) $$\tau =\frac{2\left(N_{p}-N_{q}\right)}{N(N-1)}$$

where for the joint node tuple pairs $\left(X,Y\right)=\{\left(x_{1},y_{1}\right),\left(x_{2},y_{2}\right),\ldots ,\left(x_{n},y_{n}\right)\}$ of sequences *X* and *Y*, $N_{p}$ represents the number of node pairs with the same rank order, $N_{q}$ represents the number of node pairs with different rank orders, and *N* represents the total number of nodes. The Kendall coefficient τ ranges from [−1,1]. When τ is −1, it indicates that the rankings of sequences *X* and *Y* are completely negatively correlated; when τ is 1, it indicates that the rankings of sequences *X* and *Y* are completely positively correlated.

**Figure 8:**
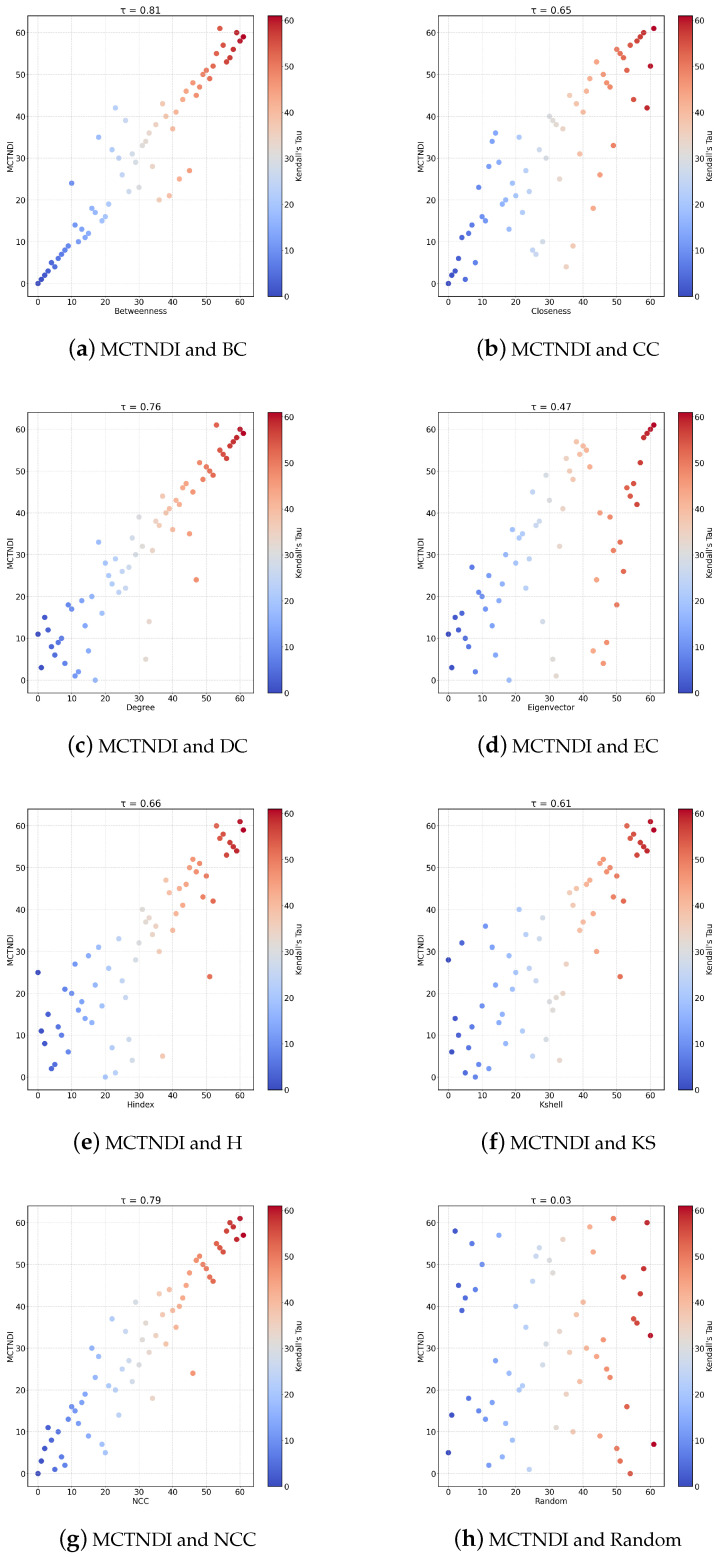
Correlation of MCTNDI and each indicator.

The correlations between MCTNDI and common single indicators for the Dolphins network are plotted in the experiments, as shown in Figure 8a–h. The horizontal axis represents the importance ranking of the nodes under a single indicator, and the vertical axis represents the importance ranking of the nodes under MCTNDI. To further analyze the similarity between indicators, we introduce degree centrality (DC), eigenvector centrality (EC), K-shell (KS), and a random indicator (Random) for the experiments.

As can be seen from Figure 8, degree centrality, betweenness centrality, and network constraint coefficient exhibit a strong positive correlation with MCTNDI, while the correlation with eigenvector centrality, K-shell, and Random is weak. Since degree centrality, betweenness centrality, and the network constraint coefficient correspond to neighborhood centrality, path centrality, and node mutual information, respectively, MCTNDI excels in capturing local network connectivity and processing node transmission and interaction information. The Random strategy ranks the nodes based on a random principle, which is quite different from the evaluation perspective of the MCTNDI. Therefore, the correlation between the ranking results of the two is weak. The previous section illustrated that the K-shell and H-index are flawed in the node importance ranking problem, so they have a weak correlation with the ranking results of the MCTNDI indicators. The H-index participates in the calculation of the node importance score as a sub-indicator of MCTNDI. However, the correlation between the two is weak, indicating that the MCTNDI is able to avoid the negative impact of such flawed indicators, then successfully extract valuable information.

## 5. Discussion

In this paper, we propose a multi-attribute decision-making indicator called MCTNDI to determine the importance of nodes in a network. This indicator integrates the CRITIC and TOPSIS methods, leveraging multiple single indicators. It not only considers the local neighborhood importance and path centrality of nodes but also combines the network topology location of nodes with node mutual information. This approach solves the issue of the single evaluation angle in most current identification methods. In MCTNDI, we first select single node importance indicators as multi-attribute decision-making sub-indicators from different perspectives. Then, the CRITIC method is used to determine the weights of each single indicator. Finally, the node importance rankings are obtained by applying weights to the decision matrix within the TOPSIS method. In the experiment, we designed corresponding experiments to answer the four research questions to confirm its effectiveness in identifying critical nodes in complex networks.

This paper conducts research in the field of complex networks and proposes an indicator for identifying critical nodes, which helps to expand the research ideas in this area. However, the indicator still has some limitations and may be restricted when applied to weighted, directed, or dynamic networks. In future research, we will explore more complex node importance indicators and extend the indicator to multi-layer networks [42] as well as dynamic networks [43] in order to be more applicable to contemporary complex real-world networks. In addition, we will explore the related techniques of graph neural networks [44] to utilize AI in empowering complex networks and continue to study node importance identification techniques applicable to variable real-world networks.

## Figures and Tables

**Table 1:** Value of indicators for each node in Figure 1.

DC		BC		CC		KS		H		NCC	
**Node**	**Value**	**Node**	**Value**	**Node**	**Value**	**Node**	**Value**	**Node**	**Value**	**Node**	**Value**
7	0.6667	8	0.3889	4	0.6429	1	3	7	4	8	0.4311
4	0.5556	4	0.2315	5	0.6429	2	3	1	3	4	0.4451
5	0.5556	5	0.2315	7	0.6000	3	3	2	3	5	0.4451
1	0.4444	9	0.2222	8	0.6000	4	3	3	3	7	0.4702
2	0.4444	7	0.1019	1	0.5294	5	3	4	3	9	0.5000
3	0.3333	1	0.0231	2	0.5294	6	3	5	3	1	0.5412
6	0.3333	2	0.0231	3	0.5000	7	3	6	3	2	0.5412
8	0.3333	3	0.0000	6	0.5000	8	2	8	2	3	0.6641
9	0.2222	6	0.0000	9	0.4256	9	1	9	1	6	0.6641
10	0.1111	10	0.0000	10	0.3103	10	1	10	1	10	1.0000

**Figure A1:**
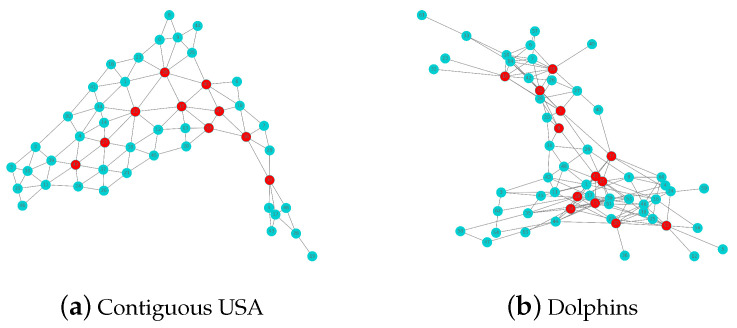

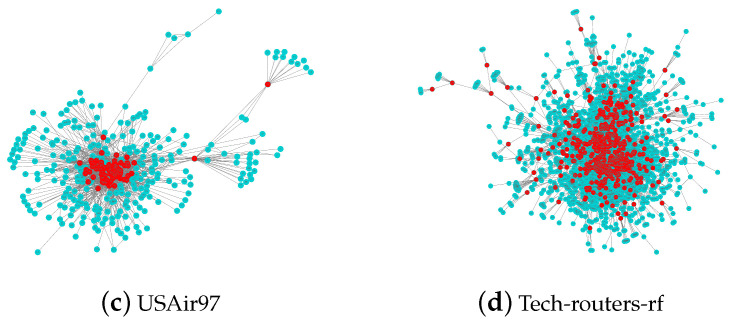
Nodes with the top 20% score in the experimental network under MCTNDI.

## Data Availability

The dataset used in this paper can be found here: https://networkrepository.com/contiguous-usa.php; https://networkrepository.com/dolphins.php; https://networkrepository.com/USAir97.php; https://networkrepository.com/tech-routers-rf.php. Four datasets were accessed on 6 October 2024.

## Appendix A

We labeled the nodes that scored in the top 20% of the four experimental networks using MCTNDI to more intuitively illustrate how MCTNDI identifies critical nodes in complex networks, as shown in Figure A1.
